# The link between nutritional parameters and bone mineral density in women: results of a screening programme for osteoporosis

**DOI:** 10.1186/1479-5876-12-46

**Published:** 2014-02-19

**Authors:** Theodora Lamprinoudi, Elisa Mazza, Yvelise Ferro, Simona Brogneri, Daniela Foti, Elio Gulletta, Maurizio Iocco, Carmine Gazzaruso, Stefano Romeo, Arturo Pujia, Tiziana Montalcini

**Affiliations:** 1 Clinical Nutrition Unit, Department of Medical and Surgical Science, University Magna Grecia, Catanzaro, Italy; 2 Clinical Pathology Unit, Department of Health Science, University Magna Grecia, Catanzaro, Italy; 3 Physiatry Unit, Department of Medical and Surgical Science, University Magna Grecia, Catanzaro, Italy; 4 Diabetes, Endocrine-metabolic Dis. Cardiovasc. Prevention Unit, Clinical Inst. “Beato Matteo”, Vigevano, Italy; 5 Department of Internal Medicine, I.R.C.C.S. Policlinico San Donato Milanese, San Donato Milanese, Italy; 6 Department of Molecular and Clinical Medicine, Sahlgrenska Center for Cardiovascolar and Metabolic Research, University of Gothenburg, Göteborg, Sweden; 7 University Magna Grecia, Viale S. Venuta, 88100 Catanzaro, Italy

## Abstract

**Background:**

A positive association between handgrip strength and bone mineral density was demonstrated, but not all the investigations confirmed these results. We conducted a screening programme for osteoporosis in a large cohort of postmenopausal women to investigate the relationship between handgrip strength, other nutritional parameters and bone density.

**Methods:**

This investigation involved 1,300 white volunteers. All participants underwent a bone mineral density evaluation at the heel and a handgrip strength measurement.

**Results:**

The mean T-score value was -1.15 ± 1; a total of 181 participants reported at least one osteoporotic fracture. In the univariate analysis, both handgrip strength and body mass index were associated with the T-score value. Adjustment for confounding factors confirmed this relationship showing, in the multivariate analysis, that the body mass index was positively correlated to the T-score (B = 0.034; p = 0.001) and, in the logistic regression analysis, that handgrip strength was associated with the presence of osteoporosis (P = 0.005).

**Conclusion:**

Both body mass index and handgrip strength were strongly correlated to bone mineral density, assessed with ultrasound, suggesting a possible key role as bone disease predictors.

## Introduction

Aging *per se* is a well-known risk factor for osteoporosis, frailty, sarcopaenia and atherosclerosis [1,2]. The mechanisms that underlie the onset of these conditions are still unclear, however, hormonal alterations, which are the main age-associated changes, are probably the key factors, also influencing comorbidity [3]. Furthermore, the increased synthesis and secretion of adipokines and other factors of inflammation associated with aging and menopause could contribute to the development of chronic diseases [4]. Comorbidity must be taken into consideration in menopause and aging, especially to develop more effective treatments to ensure well-being during the postmenopausal period. In this regard, it has been shown that handgrip strength and bone mineral density (BMD) are positively associated [5-13] suggesting a connection between sarcopaenia and osteoporosis. However, not all the investigations confirmed this result [14-18]. Consequently, we conducted a screening programme for osteoporosis in a large cohort of women to investigate the relationship between handgrip strength and BMD assessed at the heel, as well as the relationship between the ultrasound and dual energy X-ray absorptiometry (DXA) BMD assessment in postmenopausal women.

## Materials and methods

This survey was conducted from May 2012 to May 2013 at the University Hospital of Catanzaro, Italy, and involved 1,300 consecutively white volunteers of both genders. All subjects aged over 45 living in the city of Catanzaro were invited by newspapers ads to participate in the study. All participants underwent a brief interview to provide information about current and past physical exercise activity, use of medications, age at menopause and history of fractures. Pathological or high-energy fractures and fractures in sites not commonly associated with osteoporosis were not considered in the statistical analysis. Men were also excluded from statistical analysis. Postmenopausal status was defined as the presence of a serum follicle-stimulating hormone (FSH) level of over 40 IU/l (if available) or no natural menses for at least 1 year. All participants underwent a BMD and handgrip strength measurement. We obtained their informed consents to participate in the study. The investigation conforms to the principles outlined in the Declaration of Helsinki.

### Handgrip strength measurement

The handgrip strength was measured by dieticians previously trained in the technique. Subjects unable to perform the strength measurements for any cause were excluded from the statistical analysis. The handgrip strength was measured using an hydraulic hand dynamometer (Hersteller/manufactures; SAEHAN Corporation, Masan- Korea; Distributor Rehaforum Medical GmbH, Elmshorn- Germany) having less than 10% variation in results for various grip positions. Subjects were seated, with their elbows flexed at 90° and supported at the time of the measurement. Dieticians collected three measurements from each hand, and used the mean value in all analyses. During the measurement, we asked the subject to grip the dynamometer with maximum strength, and to hold the grip for at least three seconds [19-21]. Handgrip strength is registered as maximum kilograms of strength applied during the registration.

### Ultrasound BMD assessment

Quantitative ultrasound (Lunar Achilles Insight, GE Medical Systems) was used to measure the speed of sound (metres per second) and broadband ultrasound attenuation (decibels per megahertz) of the heel. In cases of a previous fracture within the lower extremity, only the opposite calcaneus was measured. T-score was derived from the value of broadband ultrasound attenuation and expressed as the number of SDs from the mean value of a control gender-matched population [22]. The T-scores are reported as the number of standard deviations below the young adult mean (normal, > -1; osteopaenia, -1 to -2.49; osteoporosis, ≤ -2.5) [22]. The device was calibrated daily in accordance with the manufacturer’s recommendations. The same operator took all the measurements. Short-term *in vivo* precision was established on the basis of repeated measurements in 10 healthy women. The coefficient of variation (CV%) was calculated using the following formula: CV% _ (SD/mean) 100; the percentage value was: 4% for broadband ultrasound attenuation and 1.2% for speed of sound.

### DXA assessment

Postmenopausal women with a high risk of fractures [5,14] who also gave their informed consent underwent a concomitant BMD of the lumbar spine and left femur evaluation by DXA. BMD was expressed as the amount of mineral (g) divided by the area scanned (cm^2^). All DXA measurements were performed by the same densitometer (Lunar DPX BRAVO, GE Medical Systems, Madison WI) and operator. Low BMD was defined according to the T-score, calculated on the basis of the normal reference values. The T-scores are reported as the number of standard deviations below the young adult mean (normal, > -1; osteopaenia, -1 to -2.49; osteoporosis, ≤ -2.5) while the Z-score was the number of standard deviations in comparison to women of the same age [22].

The instrument was calibrated every day in accordance with the manufacturer’s recommendations. The *in vivo* precision was established on the basis of repeated measurements in 40 women and was <1%.

### Statistical analysis

Data are reported as mean ± standard deviation (SD). A chi square test was performed to analyse the prevalence. A *T*-test was performed to compare the means between women with and without osteoporosis. The Pearson correlation was used to identify the variables correlated to the T-score (the number of SDs) obtained from the value of broadband ultrasound attenuation (BUA T-score) given that the continuous variables were normally distributed. The Multivariate linear regression analysis was used to test the association for confounding variables selected from univariate analysis having a p <0.1. Furthermore, a logistic regression analysis was used, considering the presence of osteoporosis as a dependent variable, and including, as independent variables, all those significantly different in the *T*-test and those associated with BUA T-score in the univariate analysis. The area under the receiver operating characteristic (ROC) curve was used to analyse the capacity of the ultrasound screening test on the heel, to predict the normal BMD with DXA evaluation in a group of postmenopausal women receiving a concomitant DXA evaluation.

Significant differences were assumed to be present at p < 0.05 (two-tailed). All comparisons were performed using SPSS 20.0 for Windows (S. Wacker Drive, Chicago, Illinois 60606, USA).

## Results

Complete data were obtained from 1,058 women. The mean BUA T-score was -1.15 ± 1.2; 181 participants experienced at least one osteoporotic fracture [specifically n = 94 of the wrist, n = 57 of the lower limbs (including femur neck), n = 39 of the vertebrae and ribs]. None of the participants had received medication for rheumatoid arthritis or oral steroids for a prolonged period of time. The prevalence of osteoporosis [22] was 16% and 90% were postmenopausal. The characteristics of the whole population are shown in Table 1. Table 2 shows the factors associated with the BUA T-score. The Multivariate linear regression analysis (Table 3) showed that only age and BMI were correlated to the BUA T-score; Handgrip Strength did not remain in correlation in this analysis. The comparison between women with and without osteoporosis (Table 4) showed a significant difference in age, Handgrip Strength, and in the age of menopause. The logistic regression analysis showed that age and Handgrip Strength were correlated to the BUA T-score (Table 5). However, when we divided the population according to the presence of fractures, women with fractures had a higher age and a lower BMD than those without fractures (p < 0.001), while there was not a significant difference in the anthropometric parameters between the two groups (data not shown). A group of 62 postmenopausal women underwent a concomitant DXA evaluation. In these participants the area under the ROC curve for BUA T-score to predict DXA measurement was 0.705 (SE = 0.076; p 0.020). The BUA T-score equal to -3 achieved acceptable sensitivity (77%), but the corresponding specificity remained poor (50%); while a BUA T-score equal to -2.7 achieved a minor sensitivity (58%), with a satisfactory corresponding specificity (72%).

**Table 1:** Demographical and clinical characteristics of the whole population

**Variables**	**Mean**	**SD**
Age	57,2	10,4
Age at menopause	48,9	5,1
BMI	27,5	4,9
BUA T-score	-1,15	1,2
DXA T-score (femur)*	-1,74	0,9
DXA T-score (vertebrae)*	-1,92	1,1
Handgrip (kg)	24,16	6,1
Prevalences		
Menopausal %	90	
Normal BMD %	39	
Osteopenia %	45,5	
osteoporosis %	16	
Diabetes %	7	
hyperlipidemia %	17	
Hypertension %	40,4	
With CVD %	16,7	
Total fractures %	26	
Medications		
Calcium %	5,7	
Vitamin D %	8,5	
Antiresorptive agents %	6,4	

*Only in a subgroup.

**Table 2:** Univariate analysis- Pearson correlation

**Factors**		**Age**	**Age at menopause**	**Handgrip**	**BMI**
**Variable**	Pearson correlation	-0,358	0,025	0,201	0,115
**US T- score**	p	<0,001	0,487	<0,001	0,009

**Table 3:** Multivariate Linear regression analysis- factors correlated to BUA T-score

**Variables**	**B**	**SE**	**beta**	**t**	**p**
**Age**	-0,046	0,006	-0,335	-8,092	<0,001
**BMI**	0,034	0,010	0,137	3,318	0,001

Excluded variable: Handgrip = beta 0,052; p = 0,245.

**Table 4:** Comparison between women with and without osteoporosis

	**Without osteoporosis**		**With osteoporosis**		**p**
**Variables**	**Mean**	**SD**	**Mean**	**SD**	
*t-test*					
**Age (ys)**	56,40	10,104	58,78	11,007	0,001
**Age at menopause (ys)**	49,09	4,977	48,13	5,559	0,020
**Hand grip strenght (kg)**	24,57	6,130	22,94	5,940	<0,001
**BMI**	27,3232	4,83373	28,1585	5,58306	0,109
*Chi square test*					
**Total fractures (%)**	24		37		<0,001

**Table 5:** Logistic regression analysis - factors correlated with Osteoporosis

	**B**	**SE**	**p**	**exp(B)**
**Age**	0,049	0,019	0,011	1,050
**Age at menopause**	-0,027	0,025	0,284	0,973
**Handgrip**	-0,054	0,027	0,050	0,948

## Discussion

From this survey we obtained several relevant data: first we found, in this geographical area, a prevalence of osteoporosis [22] of 16%, comparable to other investigations, and about half of population had osteopaenia, but less than 7% of screened women were medically treated for osteoporosis, in contrast with the women described in the other studies [23]. Another important aspect of this study was that BUA T-score was positively correlated to some nutritional parameters like Handgrip Strength and BMI (Table 2) also after adjustment for confounding factors in the multivariate and logistic regression analysis (Tables 3 and 5) suggesting the importance of measuring them to predict the low BMD.

These findings are very important since it is well accepted that the early phases of osteoporosis present little or no symptoms thus it is often difficult to identify women needing treatment for osteoporosis before their first fracture. These results may suggest the importance of promoting screening programmes using a non-invasive evaluation of BMD for the early identification of women with a low BMD [24,25].

Furthermore, our results confirm the role of BMI and muscular strength as key indices which may be useful as predictors for osteoporosis. The decline in muscle strength is considered a consequence of aging [26] due to the degeneration of muscle fibres. This was confirmed in our investigation, since both women with and without osteoporosis had a lower handgrip strength value compared to younger women evaluated in a previous study [21]. In addition, it has been shown that there are substantial inter-individual differences in the rate of strength decline, due to variation in genetics and environmental influences [27]. In this context, it is not clear if there is a site-specific effect of muscular strength on BMD [28]. Indeed, it has been shown that quadriceps strength can explain a large part of the association between lean mass and BMD at the femoral neck site but not at the lumbar spine site [28]. Furthermore, inflammation, hormonal changes and some chronic diseases could be determinants in the development of osteoporosis [29,30]. At this moment, the specific role of regional muscular strength on BMD is not fully clear. However, our results could contribute to add further information to this issue. Handgrip strength is probably a good index of frailty and also of some nutrient deficiencies, thus, the link with a low BMD is plausible [31]. In this study we showed a positive relationship between BMD and BMI. This concurs with other studies which showed higher BMD in obese women than in normal-weight ones, since obesity exerts a positive effect on BMD due to mechanical load and/or for conversion of androgen into oestrogen in adipose tissue [32].

Our results also raise concerns over the cost-effectiveness of such a screening intervention. Of course fracture prevention at an early stage of osteoporosis is preferable, and an osteoporosis screening programme with US may be considered more cost-effective in comparison to DXA assessment. We believe that quantitative ultrasound of the heel, in combination with the risk factors for osteoporosis assessment, is an acceptable option to DXA measurements in osteoporosis detection.

There are limitations to our study in that it was conducted in a single geographical area. Furthermore, we did not include specific measures of physical activities.

## Conclusion

In our survey, we showed that BMI and handgrip strength were strongly correlated to BMD measured with quantitative ultrasound of the heel. Despite the appreciable prevalence of total fractures in the women with osteoporosis less than half of them were medically treated in secondary prevention. Since the correlation between BMD assessed with US and that measured with DXA in postmenopausal women, our results show that there is still a need to promote screening programmes to identify those with low BMD with the aim of reducing the recurrence of fractures.

### Summary

We conducted a screening programme for osteoporosis in a large cohort of postmenopausal women to investigate the relationship between handgrip strength and bone mineral density assessed at the heel. Furthermore, we studied the relationship between bone density assessed with ultrasound and that assessed with dual energy X-ray absorptiometry.

## Competing interest

The authors declare that they have no competing interest.

## Authors’ contributions

TM and AP were responsible for study design, data analysis, manuscript writers; SR contributed to interpretation of the data, TL was responsible for enrollment, BMD evaluation and integrity of data; EM, YF, SB performed anthropometric measurement, data collection; DF, EG, MI and CG revised study design, manuscript and approved final version. All authors read and approved the final manuscript.
